# Effectiveness and analysis of factors predictive of discharge to home in a 4‐year cohort in a residential transitional care unit

**DOI:** 10.1002/agm2.12076

**Published:** 2019-09-08

**Authors:** Daniel Kam Yin Chan, Shouzi Zhang, Yvonne Liu, Ciaran Upton, Priya Elsa Kurien, Rui Li, Mark I. Hohenberg, Wai Tak Hung

**Affiliations:** ^1^ University of NSW Bankstown‐Lidcombe Hospital Bankstown NSW Australia; ^2^ Beijing Geriatric Hospital Beijing China; ^3^ Western Sydney University School of Medicine Penrith South DC NSW Australia; ^4^ University of Technology Sydney Sydney NSW Australia

**Keywords:** community, effectiveness, predictors, post‐acute, residential, transitional

## Abstract

**Objective:**

The aim of this study was to evaluate the effectiveness and identify factors predictive of home discharge in a cohort of patients admitted to the residential Transitional Aged Care Program (r‐TACP) after a stay in an acute hospital.

**Methods:**

A retrospective observational cohort study of patients admitted to a single r‐TACP unit between 1 January 2014 and 31 December 2017 was carried out. Baseline patient characteristics and discharge outcomes were analyzed.

**Results:**

Three hundred sixty‐nine patients were admitted during the study period. The discharge outcomes were as follows: 68% returned home, 17% went onto residential care, 14% were readmitted to hospital, and 1% died. Factors associated with not returning home were increased age, increased comorbidities, and lower Barthel Index on admission to the r‐TACP.

**Conclusion:**

Our r‐TACP is an effective program that successfully returns the majority (67.8%) of older patients home after an acute hospital admission. Older patients with greater comorbidities and poorer baseline functional status in our program were less likely to return home.

## 1 INTRODUCTION

In Australia, the Transitional Aged Care Program (TACP; also known as “TCP”) is a form of short‐term, post‐acute rehabilitation and care service provided to people aged 65 years or older at the end of an acute hospital stay. It was established in 2005 by the government and provides a time‐limited (maximum of 12 weeks), goal‐oriented, flexible package of services that helps the transition of patients from hospital to home. It includes a range of services (in different combinations according to patients’ needs), namely, physical therapy and personal or home care services provided by a multidisciplinary team aiming at optimizing functional capacity and/or patients’ home situations. It targets older patients who still require more time to restore their function to the point of being able to cope fully at home and would otherwise have required residential care prematurely.^1^

If the TACP is delivered at a recipient's home, it is called the “community Transitional Aged Care Program (c‐TACP).” Sometimes, frailer patients who are not suitable for the c‐TACP may have the alternative of receiving it at a residential facility; in that case, it is called the “residential Transitional Aged Care Program (r‐TACP).”

A brief report consisting of small numbers from the c‐TACP (59 patients) and r‐TACP (30 patients) was published initially; however, the samples had been collected from three different services. With heterogeneity and a small sample size, the authors could not draw any definitive conclusion.^2^

Subsequently, a national evaluation summarized 23 reports (2443 patients) on the TACP operation completed between September 2006 and May 2007^1^ and concluded that older patients who received TACP were deemed to be frail, with 37% returning to hospital at least once within 3 months, and 47% within 6 months. However, the report did demonstrate reduction of residential aged care facility (RACF) placement and hospital re‐presentation rates at 6 months for those who used the TACP compared with historical controls. Of note is that the report grouped both c‐TACP and r‐TACP together in its analysis and there was heterogeneity in the services model (49% received c‐TACP, 42% received r‐TACP, and 9% received both), making it difficult to conclude definitively.^1^ While theoretically, this is a service that could reduce the length of hospital stay, reduce cost, and help patients return home, few reports in the Australian setting have rigorously evaluated these salient outcomes.^3^, ^4^

Although there are a few other studies assessing outcomes of similar programs, these outcomes varied. Different patient characteristics and service models might have contributed to the differences in results and, importantly, none have reported on an r‐TACP.^5^, ^6^, ^7^

Summing up, despite the small number of studies published formally and informally to date, the results produced have been conflicting regarding the utility of TACPs. Importantly, there have been no published consensus data about patient selection into TACPs that would lead to desirable outcomes; this is especially the case for the r‐TACP, a place where frail older patients are preferentially discharged instead of to the c‐TACP. As Parsons et al^6^ have pointed out, there is a paucity of information about what type of patients would benefit most with desirable outcomes, such as “returning home” or “not being readmitted to hospital.” Allen et al^8^ in their systematic review of the c‐TACP also noted the variability of outcomes and service models, and we note that there is no r‐TACP study included in their review.

With this background, we set out to study if patients’ baseline characteristics would be good predictors for desirable outcomes (returning home, not readmitted to hospital) in a single residential TACP center.

## 2 OBJECTIVE

### 2.1 Primary objective

Our primary objective was to evaluate the effectiveness and identify the baseline factors associated with successful discharge home from the r‐TACP unit.

### 2.2 Secondary objective

Our secondary objective was to evaluate the length of stay in the r‐TACP unit and the improvement in function of the r‐TACP cohort.

## 3 METHODS

### 3.1 Study design and service model

This was a retrospective study assessing 4 years’ data set up since the inception of the r‐TACP in metropolitan South Western Sydney. The r‐TACP unit consists of 13 beds, based within a nongovernment‐run aged‐care facility (at hostel level) that also provides respite and permanent care for other residents. The r‐TACP unit is located separately from the other services and is staffed with designated on‐site nursing and part‐time allied health as well as being serviced by three‐times‐weekly geriatrician visits.

The service is a subacute model and provides r‐TACP to hospitals in the South Western Sydney Local Health District. To enter into the r‐TACP unit, hospital patients require prior approval by the Aged Care Assessment Team, which assesses if referred patients are appropriate. They are then referred onto the r‐TACP care coordinator who facilitates the transfer. Patients are excluded if they are medically unstable, require intravenous therapy, supplemental oxygen, or two‐people assistance for mobility or personal care. Patients who deteriorate medically during r‐TACP unit stay are transferred back to hospital for treatment.

In our local health district, there exists another TACP model, namely, the c‐TACP, whereby patients receive rehabilitation services from a multidisciplinary team at their own homes (following acute illnesses) instead of a residential facility. Patients are usually less frail but they also require prior approval from the Aged Care Assessment Team. Patients from the two models are serviced by separate teams and not mixed together.

There are 13 beds in our r‐TACP unit with nursing staffed at the hostel level and salaries paid by the nongovernment organization that owns the facility. In addition, the rehabilitation is serviced by a multidisciplinary team led by a part‐time geriatrician and paid for by our Health Department (Table 1).

**Table 1:** Bankstown r‐TACP unit staff hours

	No.	Hours per day	Day	Total per week
Care staff				
am	2	7.5	7	105
pm	2	7.5	7	105
Night	1	8.5	7	59.5
Registered nurse	1	7.5	7	52.5
Occupational therapist	2 (P/T)	3.3	3	10
Physiotherapist	1	3	3	9
Care manager	1	7.5	7	52.5
Administration	1	7.5	7	59.5
Social worker	1 (P/T)	5	3	15
Physiotherapy aid	1	7.5	7	52.5
Geriatrician	1 (P/T)	4	3	12

Abbreviation: P/T, Part‐time.

The r‐TACP unit provides a 1‐hour, group exercise session each morning led by the physiotherapist. In addition, there is a personal (individual) extra session of 2‐3 hours each week for those patients who need it. Regarding occupational therapy, each person gets an average of 3 hours of assessment/training time plus a home visit in cases that require it. All are screened by the social worker, and for those who need further time, up to 1 hour per week is spent, which includes family conferences.

### 3.2 Patients’ data collection

All patients admitted to the unit between 1 January 2014 and 31 December 2017 were identified from the preexisting r‐TACP electronic database. Two reviewers (S. Z. and Y. L.) reviewed all patients’ data and retrieved relevant information from the database. S. Z. did the preliminary data collection, which was counterchecked by Y. L., and any discrepancy was reconciled under the supervision of C. U. and D. K. Y. C. Study data were extracted from the database. Where there were missing data, hospital records were reviewed by Y. L. These data included: presence of delirium in hospital; baseline patient characteristics, including age, sex, and living situation prior to admission; comorbidities for Charlson Comorbidity Index (CCI); and cognitive test scores (Mini‐Mental State Examination, Rowland Universal Dementia Assessment, or Montreal Cognitive Assessment). Other comorbidities were also assessed, including history of falls in preceding 12 months, dementia, depression, and active cancer. The main reasons for hospital admission prior to r‐TACP were categorized into fractures, musculoskeletal complaints without fracture, falls, stroke (ischemic or hemorrhagic), and other medical illnesses. Functional status was measured by the Barthel Index (BI) on admission to and on discharge from the r‐TACP unit. BI was scored as zero pragmatically for patients who were readmitted to hospital or who died. Ethics approval from this study was granted by the Local Health District Research and Ethics Committee (HREC reference LNR/17/LPOOL/541).

### 3.3 Discharge destination and other patient outcomes

The main outcome measures of this study were factors associated with home as the discharge outcome at the end of the r‐TACP unit stay. There were four discharge outcomes: returning home, readmission to hospital, discharge to RACF, or death. Other study outcomes were length of stay at the r‐TACP unit and change in BI on discharge from the r‐TACP unit compared to on admission to the r‐TACP unit.

### 3.4 Statistical analysis

All collected data were recorded into a Microsoft Excel 2010 spreadsheet. The data were analyzed using SPSS 23 (IBM SPSS Statistics), GraphPad Prism 7.03 (GraphPad Software), and R 3.4.2 (https://www.r-project.org). Continuous variables were presented as mean values with standard deviation and an independent samples *t* test was used to compare the mean for the group of patients who returned home after discharge with that of all other patients. Nonparametric Fisher's exact test was used to compare the categorical data between the two groups. We performed univariate logistic regression and selected factors that have *P*‐value <.2 for the backward stepwise logistic regression. This method would identify factors predictive of returning home. A *P*‐value of <.05 was considered statistically significant.

## 4 RESULTS

### 4.1 Patient characteristics

A total of 369 patients were admitted to the r‐TACP unit during the study period. Overall, the mean age was 82.9 ± 7.84 years. There were 239 (64.8%) women and 130 (35.2%) men. Over half of the patients had lived alone prior to hospital admission. In total, 77.5% of the patients had had a history of falls in the preceding 12 months, 20.1% had had delirium during hospital admission, 23.6% had had cognitive impairment, and 10.0% had had a diagnosis of dementia. Also, 20.9% had a history of depression and 6.2% had active malignancy at the time of admission to the r‐TACP unit. The average CCI score was 2.5 ± 2.09. Cognitive test scores were recorded for just 154 (41.7%) of the patients and a variety of instruments had been used, including the Mini‐Mental State Examination, the Rowland Universal Dementia Assessment Scale, and the Montreal Cognitive Assessment. Table 2 summarizes the baseline characteristics of the patients.

**Table 2:** Baseline patient characteristics

Characteristics	Overall	Return home	Others (hospital readmission, RACF, or death)	*P*‐value
Demographics				
Overall, n (%)	369 (100)	250 (67.8)	119 (32.2)	—
Age, mean (SD)	82.9 (7.8)	82.1 (8.1)	84.3 (7.2)	.022
Female sex, n (%)	239 (64.8)	167 (66.8)	72 (60.5)	.237
Previously living alone, n (%)	215 (58.3)	144 (57.6)	71 (59.7)	.707
Comorbidities				
History of falls, n (%)	286 (77.5)	192 (76.8)	94 (79.0)	.637
Delirium during hospital admission, n (%)	74 (20.1)	48 (19.2)	26 (21.8)	.553
Cognitive impairment, n (%)	87 (23.6)	54 (21.6)	33 (27.7)	.195
Dementia, n (%)	37 (10.0)	20 (8.0)	17 (14.3)	.060
Depression, n (%)	77 (20.9)	54 (21.6)	23 (19.3)	.616
Active cancer, n (%)	23 (6.2)	14 (5.6)	9 (7.6)	.466
CCI, mean (SD)	2.5 (2.1)	2.2 (2.0)	3.0 (2.2)	.002
Functional status				
Baseline BI, mean (SD)	64.0 (58.7)	66.8 (11.5)	63.7 (11.3)	.015
Final BI, mean (SD)	86.5 (10.7)	87.9 (9.7)	81.4 (12.6)^a^	<.001
Change in BI, mean (SD)	20.3 (12.5)	21.1 (12.6)	17.3 (11.6)	.020
% Change in BI from baseline, mean (SD)	33.7 (24.5)	34.9 (25.2)	29.0 (21.5)	.055
Outcome				
LOS in r‐TACP, mean (SD)	46.8 (22.5)	48.3 (20.7)	43.6 (25.7)	.083
Death, n (%)	3 (0.8)	0	3 (2.5)	.012

Abbreviations: BI, Barthel Index; CCI, Charlson Comorbidity Index; LOS, length of stay; RACF, residential aged care facility; r‐TACP, residential Transitional Aged Care Program.

a Calculations excluded patients who had a default final BI score of zero, ie, those who were readmitted to hospital or who died.

### 4.2 Reasons for hospital admission

The reasons for hospital admission prior to transfer to the r‐TACP unit between the two groups (those who returned home and those who did not) are shown in Table 3. The most common diagnosis was fractures (38.5%), followed by medical illness other than falls (26.6%), falls (13.8%), and stroke (7%).

**Table 3:** Principal diagnoses for initial hospital admission

Principal diagnoses	Overall, n = 369	Return home, n = 250 (%)	No return home, n = 119 (%)
Fractures (38.5%)	142	98 (69.0)	44 (31)
Medical illness (26.6%)	98	63 (64.3)	35 (35.7)
Musculoskeletal (14.1%)	52	39 (75.0)	13 (25.0)
Falls (13.8%)	51	28 (54.9)	23 (45.1)
Stroke (7.0%)	26	22 (84.6)	4 (15.4)

### 4.3 Discharge destination and other patient outcomes

A total of 250 patients (67.8%) returned home after r‐TACP. Forty‐five patients (12.1%) were transferred to low‐level residential care, 18 (4.9%) were transferred to high‐level residential care, 48 (13.0%) were readmitted to hospital, three (1.0%) died at the r‐TACP unit, and five (1.4%) patients were discharged to another destination.

The average length of stay at the r‐TACP unit was 46.8 ± 22.5 days. Patients who returned home stayed on average 5 days longer than patients who did not return home but this did not reach statistical significance (48.3 vs 43.6 days, *P* = .083).

Functional status was measured by the baseline BI on admission to the r‐TACP unit and the final BI on discharge from the r‐TACP unit. Patients who were readmitted to hospital or died during r‐TACP were pragmatically scored a final BI score of zero in the database and were therefore excluded in the statistical analysis. Overall, the mean BI on admission was 65.8 ± 11.5 and increased to 86.5 ± 10.7 on discharge, achieving a mean gain of 20.3. Patients who did not return home had lower baseline BI and achieved smaller gains in BI on discharge compared to patients who returned home (17.3 vs 21.1, *P* = .020).

### 4.4 Baseline factors associated with home as discharge destination

Baseline factors predictive of returning home were identified from a backward stepwise logistic regression model. The base model included demographic factors of age group, CCI, presenting principal diagnoses for hospitalization, baseline BI, and dementia (Table 4). These predictors have *P*‐values <.2. The factors found to be predictive and retained in the final regression model were age group, CCI, and baseline BI (Table 5). Increased CCI (adjusted odds ratio [OR], 0.84; 95% confidence interval [CI], 0.76‐0.94; *P* = .002) was associated with lower chance of returning home. However, age group was a significant predictor of the outcome of returning home (*P* = .038). Also, patients aged <75 years had a 2.6‐times higher chance of returning home than those aged >85 years (adjusted OR, 2.6; 95% CI, 1.21‐5.59; *P* = .014). On the contrary, higher baseline BI (adjusted OR, 1.02; 95% CI, 1.00‐1.04; *P* = .039) was associated with borderline increased likelihood of returning home.

**Table 4:** Univariate analysis for baseline factors predicting return home

Univariate variable	OR (95% CI)	*P*‐value
Sex: female vs male	1.31 (0.84‐2.06)	.239
Age (reference >85 y)		.023
81‐85 y	1.19 (0.69‐2.04); *P* = .528	
75‐80 y	1.97 (1.06‐3.68); *P* = .032	
<75 y	2.60 (1.21‐5.59); *P* = .014	
Charlson Comorbidity Index	0.85 (0.77‐0.94)	.002
Baseline Barthel Index	1.02 (1‐1.04)	.015
Diagnosis (reference “falls”)		.051
Stroke	4.52 (1.36‐14.99); *P* = .014	
Musculoskeletal	2.46 (1.07‐5.68); *P* = .034	
Fractures	1.83 (0.95‐3.53); *P* = .071	
Medical illness	1.48 (0.74‐2.95); *P* = .266	
Living alone (no vs yes)	1.09 (0.7‐1.7)	.707
Delirium (no vs yes)	1.18 (0.69‐2.01)	.555
Dementia (no vs yes)	1.92 (0.96‐3.81)	.067
Cancer (no vs yes)	1.38 (0.58‐3.28)	.473
Depression (no vs yes)	1.15 (0.67‐1.98)	.614
Fall in last 12 months (no vs yes)	1.14 (0.67‐1.93)	.636

Abbreviations: CI, confidence interval; OR, odds ratio.

**Table 5:** Baseline factors predictive of discharge outcome after multivariate logistic regression

Factors predictive of return home	OR (adjusted)	95% CI	*P*‐value
Age (reference >85 y)			.038
81‐85 y	1.07	0.62‐1.86; *P* = .808	
75‐80 y	1.99	1.05‐3.79; *P* = .035	
<75 y	2.33	1.07‐5.07; *P* = .034	
Charlson Comorbidity Index	0.84	0.76‐0.94	.002
Baseline Barthel Index	1.02	1.00‐1.04	.039

Abbreviations: CI, confidence interval; OR, odds ratio.

## 5 DISCUSSION

Out of the 369 patients in our r‐TACP over 4 years, the majority (68%) returned home. We also found that CCI was a reasonable predictor associated with homeward outcome. A mean CCI of 3 was associated with the less desirable outcome of RACF placement, while a lower mean CCI of 2.2 was associated with a homeward discharge destination. The mean gain in BI was 20.3 and this was achieved at the expense of 46.8 days’ stay in the program.

A 2008 report^1^ found that by the 3‐month follow‐up, 35% of patients had been readmitted to hospital at least once, 45% were in permanent residential care, and 14% had died. By 6 months, these numbers had increased to 43%, 58%, and 20%, respectively. Unfortunately, we do not have the follow‐up data of 3 and 6 months to compare, and even if we did, the previous data were from a decade ago, and many variables that may affect outcomes are not readily available for consideration.

The CCI was initially validated in an acute hospitalized setting to predict 1‐year mortality, with higher scores being associated with higher mortality risk.^9^ While overreliance on this simple tool to select patients for our r‐TACP may not be ideal, it does give us confidence that the current patient selection process appears to yield reasonable results (68% of patients were able to return home). If too many RACF‐bound patients are admitted, it creates patient flow challenges for hospitals as patients frequently wait in acute hospital beds until an RACF bed is available.

## 6 CONCLUSION

Our r‐TACP is an effective transitional program that successfully returns the majority of frail older patients home after an acute hospital admission. Older patients with greater comorbidities and poorer baseline functional status in our program were less likely to return home although our current selection process appears to be adequate in screening out many patients who may not benefit. The r‐TACP model of care can be considered for use in similar hospitals with similar patient demographics.

## CONFLICTS OF INTEREST

Nothing to disclose.

## AUTHOR CONTRIBUTIONS

All authors: Writing of paper. Daniel K. Y. Chan: Design, literature review, coordination. Ciaran Upton: Design, data collection. Yvonne Liu: Data collection and cleansing, review of medical records, statistical analysis. Professor Shouzi Zhang and Rui Li: Data collection, review of medical notes, initial statistical analysis. Wai Tak Hung: Data analysis, second round statistical analysis. Mark I. Hohenberg: Literature review. Priya Elsa Kurien: Data collection.
